# Using Primary care data metrics to inform policy and practice: Human Health Resource implications.

**DOI:** 10.23889/ijpds.v7i3.2051

**Published:** 2022-08-25

**Authors:** Eliot Frymire, Michael Green, Richard Glazier, Shahriar Khan, Kamila Premji, Imaan Bayoumi, Liisa Jaakkimainen, Tara Kiran, Peter Gozdyra

**Affiliations:** 1 Queens University; 2 St. Michael’s Hospital; 3 ICES Queens; 4 University of Ottawa; 5 ICES Toronto

## Objectives

To produce open access Primary Care Data Reports using standard health administrative measures in primary care in conjunction with measures for attachment to a primary care provider. Illustrate the importance of incorporating patient attachment data as an essential component in Human Health Resource (HHR) planning.

## Approach

This cohort study uses standard health administrative linked data in primary care in conjunction with measures of attachment to a primary care provider for the population of Ontario, Canada (14,632,575). Data includes attached and uncertainly attached patients stratified according to key demographics, patient characteristics, health care utilization and primary care indicators. We stratified based on health utilization characteristics and produced 6 priority populations of interest by region.

## Results

The factors most often utilized in informing human health resource planning were based on policy and practice users input and included:1.Patient enrolment model, 2.Attachment to a primary care provider, 3.Who does and does not receive care, 4.Continuity with regular source of care. Policy planners use the reports for improved understanding of the scope of issues in regions and improved understanding of primary care involvement with priority populations. Policy planners have used this report as a data support and measurement tool to identify supply (physician) and demand (patient) data essential in HHR planning. Health system reform initiatives can use this data to inform improvements in the quality of, and equitable access to, primary care services in specific jurisdictions.

## Conclusion

These reports contain key physician and patient data characteristics that correspond to primary care attachment rates. This data is essential to HHR planning when the goal is improving access to primary care for both attached and uncertainly attached patients. Data visualization in the form of mapping is especially impactful for policy and practice stakeholders.

